# In vitro biological properties and health benefits of a novel sulfated polysaccharide isolated from *Cymodocea nodosa*

**DOI:** 10.1186/s12944-017-0643-y

**Published:** 2017-12-22

**Authors:** Rihab Ben Abdallah Kolsi, Bochra Gargouri, Sameh Sassi, Donyez Frikha, Saloua Lassoued, Karima Belghith

**Affiliations:** 1 Laboratory of Plant Biotechnology Applied to the Improvement of Cultures, Faculty of Sciences of Sfax, 3038 Sfax, Tunisia; 2 Biotechnology Unit and Pathologies, Superior Institute of Biotechnology of Sfax, 3038 Sfax, Tunisia; 3 Unité de Biodiversité et Valorisation des Bioressources en zones arides, Faculté des Sciences de Gabes, Gabes, Tunisia; 4 Biodiversity Unit and aquatic ecosystems, Faculty of Sciences of Sfax, 3038 Sfax, Tunisia

**Keywords:** Polysaccharide, GCMS, DNA, PCR, Antioxidant, Antimicrobial, Cytotoxicity test

## Abstract

**Background:**

During the last few decades, there has been a growing interest in the search for novel bioactive compounds from marine origins.

**Methods:**

The present study is the first to determine the molecular characterization which it was deposited in the genebank database, to investigate and evaluate the biological properties of sulfated polysaccharide from *Cymodocea nodosa* (CNSP) seagrass.

**Results:**

The results revealed that CNSP had high activity in total antioxidant assay (59.03 mg ascorbic acid equivalents/g extract), reducing power (OD = 0.3), DPPH radical scavenging (IC_50_ = 1.22 mg/ml) and ABTS radical scavenging (IC_50_ = 1.14 mg/ml). It was also noted to exhibit antimicrobial activity against a wide range of microorganisms, with important inhibition zones. The results revealed that CNSP was able to inhibit the proliferation of Hela cell lines with a dose-dependent manner.

**Conclusion:**

Overall, the results presented in this study demonstrate that CNSP has several attractive antioxidant, antimicrobial and antiproliferative properties with potential benefits towards health.

## Background

In recent years, many marine resources have attracted attention in the search for bioactive compounds, in order to develop new drugs and dietetic foods. In fact, sulfated polysaccharides are natural substances well known for their biological properties. They act as free radical scavengers for the protection of living organisms from oxidative damage [1], antibiotics [2] and as antiproliferative agents [3]. The literature indicates that the marine environment offers a rich source of structurally diverse and bioactive polysaccharides [4].

The search of new nutritional antioxidants from marine plant sources has received an important attention in recent research. The protective effects of antioxidants derive from their capacities to (I) scavenge free radicals by acting as hydrogen/electron donors; (II) chelate ions of transition-metal; (III) inhibit free radical-producing enzymes, such as lipoxygenase, cyclooxygenase and NADPH oxidases and increase the reaction of antioxidant enzymes (superoxide dismutase (SOD), catalase (CAT) and glutathione peroxidase (GPX)) [5, 6]. Antioxidant products from natural origins have attracted special appreciation because of their free radical scavenging capabilities. Furthermore, the growing concerns over the serious problems associated with the use of conventional antibiotics and food preservatives has revived the interest in antimicrobial agents from medicinal plant origins [7].

In particular, the marine seagrasses species covering the coastal marine environment for 100 to 120 million years form complex systems with promising functional properties [8]. Only 4 Genus that are widely distributed on our Tunisian coasts represented by *Posidonia, Cymodocea, Zostera* and *Halophila.*

Although these marine species are widely distributed in the Mediterranean region but little work has been performed to explore their promising potential as a source of natural bioactive compounds for use as food additives and preservative agents in the nutraceutical and functional food industries. Given the developments in both the food and medical sectors and in the search for new natural renewable phytotherapeutic resources, it seems appropriate to demonstrate the molecular and biological properties of the *Cymodocea nodosa* marine plant.

In our previous study [9], we indicated the physico-chemical, techno-functional and structural characteristics of the sulfated polysaccharide extracted from *Cymodocea nodosa* (CNSP), which conferred on the nutritional and functional properties very sought after in the food sector. In order to complete the characterization of this polysaccharide, it would be judicious, during this work, to highlight for the first time the antioxidant, antimicrobial activity against clinical and pathogenic microorganisms and the cytotoxic effects of this plant derived polysaccharide on Hela cell lines using several tests.

## Methods

### *Cymodocea nodosa* leaves collection

*Cymodocea nodosa* (CN) was collected from coast of Chebba, this plant was then rinsed with sea water and placed in plastic bags. At the laboratory, it was rinsed again with running water and distilled water and finally dried in the open air for 72 h. It was identified at the Stazione Zoologica ‘A. Dohrn’, Functional and Evolutionary Ecology Laboratory, Punta S. Pietro, Ischia, Italy.

### Plant material

Our species was thoroughly cleaned of epiphytes and all impurities with filtered and sterilized sea water, then fragments of thallus about 0.3 g of the sample are stored at (−80 °C) until extraction and crushed with a liquid nitrogen in a mortar until obtaining a well homogeneous powder.

### Sulfated polysaccharide isolation

The methodology used for the sequential and selective extraction of this polysaccharide was adapted according to our previous study described by Ben Abdallah Kolsi et al. [9]. The extraction mode includes 3 main steps: depigmentation, extraction of the polysaccharide with hot water and a purification step.

### Chromathographic analysis

#### Acid hydrolysis

The monitoring of hydrolysis kinetics is a necessary preliminary step of defining the optimum conditions for the liberation of simple oses and oligosaccharides. The practical details of the implementation are as follows: 50 mg of the sulfated polysaccharide extract are hydrolyzed with 2 ml of 2 M trifluoroacetic acid at 100 °C for 5 h in closed tubes. After hydrolysis, the sample was neutralized with NaOH (1 M) and then centrifuged at 2000 g for 5 min. The supernatant was recovered and analyzed by thin layer chromatography (TLC) and gas chromatography-mass spectrometry (GC-MS).

#### Thin-layer chromatography (TLC)

The sugars in the reaction mixtures were analyzed by thin-layer chromatography (TLC) on silica gel type (Merck Silica Gel 20 × 20) using xylose, fructose, galactose, glucose, cellobiose, maltose, raffinose as standard monosaccharide. The mobile phase system was butanol/acetic acid/water (2:1:1 by volume). After layer development and mobile phase evaporation under continuous warm air flow for 10 min, the revelation was carried out by a mixture of H_2_SO_4_/ethanol (5, 95, v/v). Finally, a drying for 10 min at 105 °C was carried out [10].

#### Gas chromatography–mass spectrometry (GC–MS)

Determination of the composition of neutral sugars was carried out by GC-MS which has been commonly used in the analysis of the polysaccharide composition including the type and the molar ratio of the monosaccharide after hydrolysis of the CNSP with 2 M trifluoroacetic acid (TFA) at 100 °C for 5 h as indicated above. The polysaccharide solution was analyzed on an HP 5890 Series II GC chromatograph coupled to an HP 5970 mass spectrometer (Hewlett Packard, Amsterdam, The Netherlands), equipped with a fused silica (30 mx 0.25 mm) fused silica capillary column DB-225MS (Durabond) and a Varian Saturn ITD 2000 spectrometer. The temperature of the column was brought from 180 °C to 280 °C at a rate of 5 °C/min and the injector and detector are maintained at 280 °C. The carrier gas velocity used (helium) is 1 ml/min.

### Determination of in vitro antioxidant activities

#### DPPH radical scavenging activity

The test used to measure the DPPH free radical–scavenging power of the sample was that described by Brand-Williams et al. [11]. This test has the advantage of avoiding the oxidation of the substrate on which we want to test the effectiveness of an antioxidant by choosing to reduce a stable radical.

The experimental protocol was as follows: 50 μl of various concentrations of the extract to be tested are placed in the presence of 5 ml of the methanol solution of DPPH at 0.04% (OD = 0.877). After an incubation period of 30 min at room temperature, the absorbance was read against a control at 517 nm. The inhibition of DPPH free radicals is expressed as a percentage and calculated as follows:

$$ \mathrm{Scavenging}\  \mathrm{activity}\ \left(\%\right)=\left(\left({\mathrm{A}}_{\mathrm{total}}\hbox{--} {\mathrm{A}}_{\mathrm{extract}}\right)/{\mathrm{A}}_{\mathrm{total}}\right)\ \mathrm{x}\ 100 $$

The values are expressed in IC_50_ on the order of mg ml^−1^ and which represents the amount of antioxidant necessary to reduce by 50% the amount of DPPH initially present (Inhibitory concentration of 50% of radicals). The extract with the lowest IC_50_ exhibits the highest anti-free radical activity.

#### ABTS radical-scavenging activity

Evaluation of the antioxidant activity by scavenging the ABTS radical (2,2′-azino-bis-3-ethylbenzthiazoline-6-sulphonic acid) was determined according to Re et al. [12]. This method was based on the ability of antioxidants to inhibit the cationic radical of ABTS, a gray/blue chromophore with absorbance characteristics at 734 nm, in comparison with ascorbic acid.

The cationic radical of ABTS was prepared by reacting the aqueous ABTS solution with potassium persulfate which has been kept in the dark at 25 °C for 12–16 h. The solution was then diluted with ethanol or sodium acetate until an absorbance of 0.7 to 734 nm is obtained before use.

The antioxidant solution reduces the cationic radical of ABTS which results in discoloration of the reaction medium. The extinction of the discoloration was calculated as a percentage of reduction of the absorbance which was calculated as follows:

$$ \mathrm{Scavenging}\  \mathrm{activity}\ \left(\%\right)=\left(\left({\mathrm{A}}_{\mathrm{total}}\hbox{--} {\mathrm{A}}_{\mathrm{extract}}\right)/{\mathrm{A}}_{\mathrm{total}}\right)\ \mathrm{x}\ 100 $$

#### Determination of total antioxidant activity

The total antioxidant capacity of the extract was evaluated by the phosphomolybdenum method of Prieto et al. [13]. This technique was based on the reduction of Mo (VI) molybdenum present in the form of molybdate MoO_4_^2−^ to molybdenum Mo (V) MoO_2_^+^ ions in the presence of the extract to form a phosphate / Mo (V) green complex at pH acid.

A volume of 0.3 ml of the polysaccharide extract was mixed with 3 ml of the reagent solution (0.6 M sulfuric acid, 28 mM sodium phosphate and 4 mM ammonium molybdate). The tubes were screwed in and incubated at 95 °C for 90 min. After cooling, the absorbance of the solutions is measured at 695 nm against the white which contains 3 ml of the reagent solution and 0.3 ml of methanol. The total antioxidant capacity was expressed in milligrams equivalent of ascorbic acid per gram of extract (mg EAA/g extract).

#### Measurement of reducing power

The reducing power of our polysaccharide extract was determined according to the method described by Kumaran and Karunakaran [14]. 1 ml of each extract at different concentrations (0.06–1 mg/ml) was mixed with 2.5 ml of a phosphate buffer solution (0.2 M, pH 6.6) and 2.5 ml of Potassium ferricyanide [K_3_Fe (CN)_6_] (1%). After incubation at 50 °C for 20 min, 2.5 ml of a solution of trichloroacetic acid (10%) was added. After centrifugation (10 min, 1000 g), 2.5 ml of the supernatant are mixed with 2.5 ml of distilled water and 0.5 ml of FeCl_3_ (0.1%). The optical density was measured at 700 nm. The reducing power was expressed directly as a function of the optical density.

### Antibacterial and antifungal activities of CNSP

#### Microorganisms

Our study included fourteen strains of reference:

*Escherichia coli* (ATCC 8739), *Escherichia coli* DH5 (alpha), *Listeria monocytogene* (BUG 496), *Salmonella enteria* (ATCC 43972*), Agrobacterium tumefaciens, Pseudomonas aerigunosa* (ATCC 49189), *Staphylococus aureus* (ATCC6538), *Micrococcus luteus* (LB 14110), *Bacillus subtilis* (ATCC 6633)*, Bacillus amyloliquefaciens* (ATCC 6633), *Aspergilus niger*, *Saccharomyces cerevisiae*, *Fusarium oxysporum* and *Candida albicans* (ATCC 90028) were obtained from the microbiology laboratory, faculty of science, Sfax-Tunisia.

The evaluation of the antibacterial and antifungal activities was carried out by the agar diffusion method or the disk diffusion method [15].

#### Antimicrobial assay disc-diffusion method

The bacterial cultures were first grown on Muller Hinton agar (MH) plates at 37 °C for 18 to 24 h prior to seeding onto the nutrient agar. The biological activity against yeast and fungi was determined by employing disc agar diffusion method using Sabouraud Dextrose agar at 30 °C of the 24 h [16]. Consequently, each of the sterile Wattman paper disks N^°^3 and of diameter 6 mm is impregnated with 20 μl of the polysaccharide extract at a concentration of 50 mg/ml and placed on the surface of the middle of the petri dish in presence of disks impregnated with aqueous solution (negative controls). Discs of ampicillin marketed (at 10 μg/disc) as positive controls and discs of cycloheximide (10 μg/disc) which were taken as antifungal for the positive controls. The dishes were then incubated for 2 h at 4 °C and then at 37 °C for 24 h for the bacteria and at 30 °C for 48 h for the fungi. The diameters of the zones of inhibition surrounding the discs containing the samples to be tested were measured.

#### Minimum inhibitory concentration

The liquid microdilution method using Elisa plates with 96 wells was used [17]. Pre-cultures were prepared in the same manner as described above for the diffusion test. The inoculum was prepared in order to obtain a final cell density of about 10^6^CFU/ml.In the 96 well plates, serial dilutions of the extract, suitably solubilized were prepared, and an appropriate control was used as positive and negative controls. The plates thus prepared were incubated with moderate stirring at the optimum growth temperature of the microorganisms: at (37 °C) for 24 h for the bacteria and at (30 °C) for 48 h for the fungi. Following incubation, the MIC was determined by following the turn of the brick red color of the phenol to a yellow coloration in the presence of growing microorganisms. Once the growth was blocked, it was observed that the red color of the phenol persists. The MIC corresponds to the first sample concentration of the first red well with no bacterial disorder or bacterial pellet. The tests were carried out twice at the rate of 3 wells/sample during each test.

### Antiproliferative activity of CNSP

#### Cell line and culture conditions

##### Hela cell culture

The continuous human cell lines Hela (epithelial cervical cancer cell line) was investigated for cytotoxicity and antioxidant effect of CNSP. Cell sub-culture was performed every 3 to 5 days. The cells having reached the saturation concentration are centrifuged for 10 min at 1000 rpm and then suspended in 2 ml of RPMI 1640 medium (Gibco) supplemented with 10% foetal calf serum (FCS). It was passed twice a week and kept at 37 °C in a humidified atmosphere of 95% air and 5% CO_2_.

##### Preparation of cell extracts

At the end of the incubation period following treatment with the polysaccharide extract of *Cymodocea nodosa*, the cells were collected. After centrifugation for 10 min at 1000 rpm, the medium is aspirated. The cell pellet was washed once with phosphate buffered saline (PBS) and then centrifuged for 10 min at 1000 rpm. The cells were then suspended in 300 μl of PBS. Cell lysis was performed by 10 sonication cycles for 20 s.

##### Proliferation characteristics of Hela cells after CNSP treatments

Cell viability was assessed following CNSP treatments by three Bromure (4.5-diméthylthiazol-2-yl)-2.5-diphényltétrazolium; MTT test; sigma, Germany). Briefly, 2.10^6^ cells are cultured for 48 h. After treatment with 0.5; 0.25; 0.125; 0.06; 0.03; 0.015; 0.0035, 0.0015, 0.00075 and 0.00035 μg/ml of the polysaccharide extract, the cells were washed 3 times with the phosphate buffer. The cells were incubated for 48 h, 20 μl of MTT solution (0.5 mg/ml) were added to each well and incubated for 4 h. Then, 180 μl of culture medium were carefully removed from each well and replaced with 180 μl of DMSO-Methanol (1 V/1 V). The plate was then stirred until the formazan salt was completely dissolved, and then the optical density (OD) was measured by an ELISA plate reader at 570 nm.

##### Induction of oxidative stress

Cells were adjusted to 5 × 10^5^ cells / ml in 25 cm^2^ flasks and incubated at 37 °C. The oxidative stress was induced, after 72 h, by addition of Fe_2_SO_4_ to the cells at a final concentration of 100 mM, for 1 h.

##### Protein determination

Proteins were determined using the Protein Assay Kit from Bio-Rad (France) and bovine serum albumin served as the standard.

##### Lipid peroxidation determination

The malondialdehyde (MDA) assay by the TBARS method was based on the reaction between two molecules of thiobarbituric acid (TBA) with a molecule of MDA, in hot and acid medium. The result of this reaction was the appearance of a pink colored MDA- (TBA) 2 complex whose intensity is measured at 532 nm. 60 μl of plasma or of cell lysate are added to 1 ml of reagent TBA / TCA (thiobarbituric acid)/(trichloroacetic acid). The mixture was set at 95 °C for 15 min to form the MDA- (TBA) 2 complex. After centrifugation at 3000 rpm for 10 min, the MDA was assayed in the supernatant by measuring the optical density (OD) with the spectrophotometer (Biochrom, Libra S32) at 532 nm. The concentration was determined by multiplying the OD value by a regression coefficient. This was calculated from a calibration curve determined from a standard solution of 1,1,3,3-tetraethoxypropane (1,1,3,3 PET). The results were expressed in nmoles/mg of total proteins [18].

### Statistical analysis

Data were expressed by mean ± SD. Statistical analysis was carried out by analysis of variance (ANOVA) and by Student’s t-test. A *p* < 0.05 was considered to be statistically significant.

## Results and discussion

### Chemical composition and chromatographic analysis

The findings from the chemical characterization analysis presented in our previous study [9] revealed that CNSP consisted mainly of sulfate (23.17%), total sugars (54.90%) and uronic acid (11.03%) with low water activity (0.49). It had an XRD pattern that was typical for a semi-crystalline polymer with homogeneous structure, with a preliminary structural may have a backbone of branched 6-O-sulfated (1 → 4) galactosidic linkages.

The monomeric composition constituting this polymer was demonstrated by a thin layer chromatography step which revealed spots which correspond to monosaccharides obtained also by GC-MS chromatographic analysis.

**Fig. 1:**
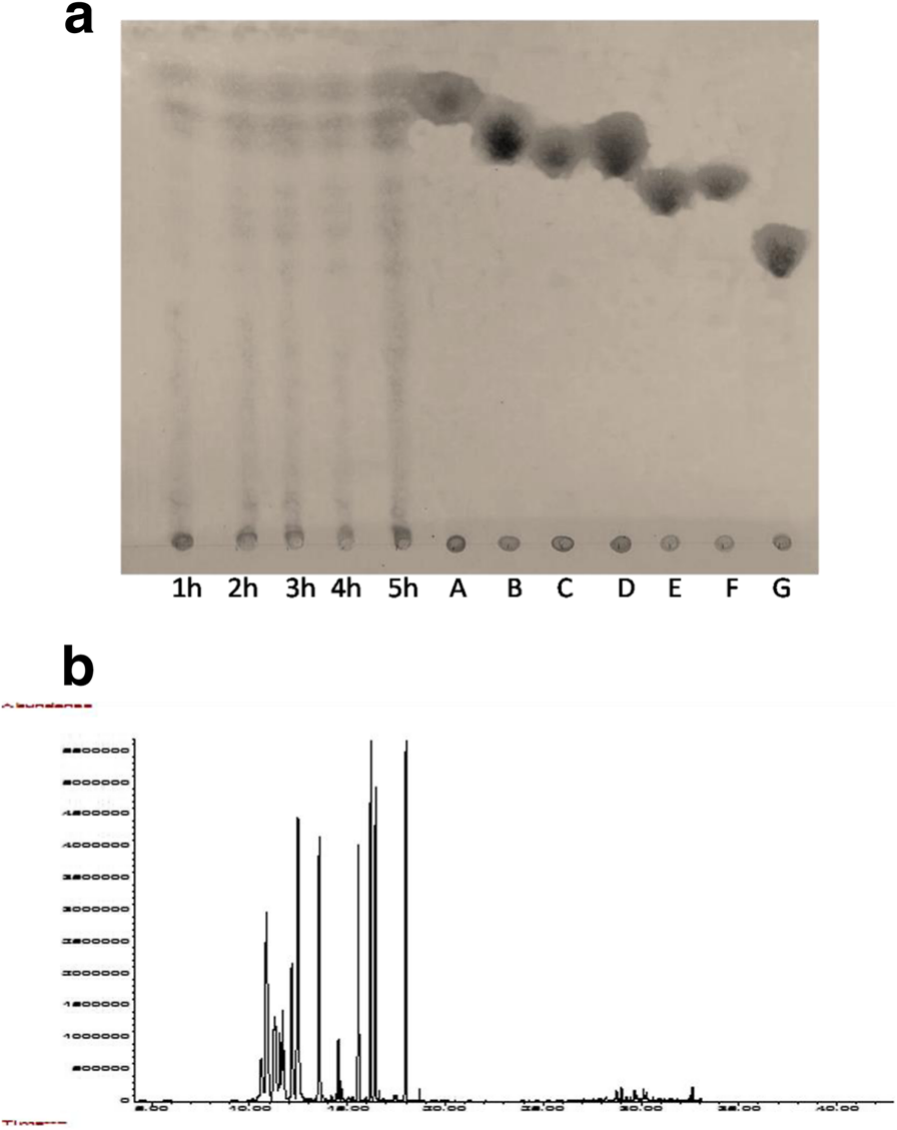
**a** TLC analysis after hydrolysis of CNSP by TFA (2 M): Reaction time (h), A: Xylose, B: Fructose, C: Galactose, D: Glucose, E: Cellobiose, F: Maltose, G: Raffinose. **b** Base peak chromatogram of *Cymodocea nodosa* sulfated polysaccharide by GC–MS.

The use of a 2 M TFA solution results in depolymerization of the polysaccharide after one hour of reaction at 100 °C (Fig. 1a).

The increase in the relative intensity of the spots between 1 h and 5 h means that an increase in the hydrolysis time is accompanied by an increased release of oligosaccharide entities. A 5 h hydrolysis time seems therefore to be a good compromise between obtaining sufficient oligosaccharides and excessive depolymerization of the native polysaccharide and the released oligosaccharides.

The evolution of the chromatographic profile as a function of time (Fig. 1a) indicates that the glycosidic bonds of the polymer exhibit various stabilities. This finding suggests that this polysaccharide is composed of a repetitive sequence that should be isolated and characterized.

The chromatogram obtained makes it possible to determine the composition of CNSP monosaccharides analyzed by GC-MS, after hydrolysis and silylation (Fig. 1b). It showed that it is a heteropolysaccharide composed of galactose (44.89%), mannose (17.30%), arabinose (12.05%), xylose (9.18%) and maltose (1.07%) at various retention times.

Overall, the results indicate that CNSP have attractive chemical composition and structural characteristics that can be related to its biological activities and pharmaceutical potential.

### Antioxidant potential

**Fig. 2:**
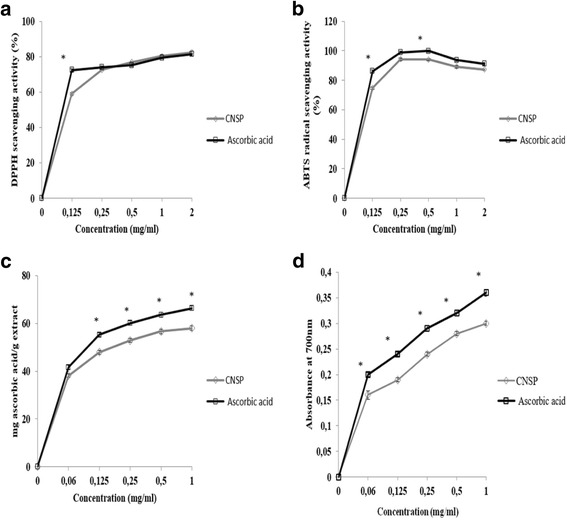
The in vitro antioxidant activities of CNSP at different concentrations. DPPH free radical-scavenging activity (**a**), ABTS radical-scavenging activity (**b**), Total antioxidant activity (**c**) and reducing power (**d**). Values are means of three replications ± SD; ^*^ *P* < 0.05 compared with different concentration of CNSP

The therapeutic benefits of natural extracts have often been attributed to their antioxidant properties. The determination of the total antioxidant potential is important because it makes it possible to evaluate the antioxidant activity of a molecule without targeting a specific free radical. The total antioxidant potential is determined by reference to a calibration curve established with ascorbic acid (Fig. 2c). This extract has an antioxidant potential that is slightly lower than that of ascorbic acid, which is a synthetic antioxidant strongly used in agro-food industries. Indeed, it varies from 38.15 to 59.03 mg ascorbic acid / g of extract and increases with increasing CNSP concentrations. However, this activity is higher than that of other sulfated polysaccharides isolated from *Ulva lactuca* and *Halodule wrightii* in the order of 9 mg ascorbic acid/g of sample and 15.21 mg ascorbic acid/g of sample respectively Which have been reported to be elevated by several authors [19, 20].

The sulfated polysaccharides of the brown alga *Sargassum swartziiet* and the red alga *Gracilaria caudata* [21, 22] exhibit a total antioxidant activity in the order of (32.34 mg ascorbic acid/g sample and 53.9 mg ascorbic acid/g sample, respectively) comparable to that reported in this work.

This difference can be attributed to the species studied, to environmental and climatic conditions (luminosity, temperature and hydrodynamic factors) and to variations in extraction and analysis methods [21, 23].

Oxidative damage caused by oxygen (hydroxyl (OH) and superoxide (O_2_) radicals on lipids, proteins and nucleic acids can cause several diseases, particularly cardiovascular diseases [24].

The determination of reducing power is another technique that can be used to better understand the mechanisms of action of antioxidants. As a result, in this study, the CNSP’s reducing power is closely correlated with that of the concentration (Fig. 2d). This observation is consistent with the results reported previously by Vijayabaskar et al. [21]. The results show that the best ability to reduce Fe^3+^ ions to Fe^2+^ is obtained at a concentration of 1 mg/ml corresponding to an OD = 0.30. These results are comparable to those obtained by the sulfated polysaccharides of the *Porphyra haitanensis*, *Ulva pertusa* and *Ulva lactuca* algae, which showed reducing powers in the order of 0.28 and 0.25 and 0.33 respectively [19, 25]. Indeed, this allows us to conclure that the CNSP reducing power is probably related to the structure of this polysaccharide having hydroxyl groups, with labile hydrogen atoms capable of neurralising the free radicals [19].

The antioxidant activity is evaluated by the method of measuring the effectiveness of the compound to be trapped in free radicals. The profiles obtained reveal that the extract of CNSP has a dose-dependent anti-radical activity (Fig. 2a). Indeed, the anti-free radical activity increases with the increase of the concentration of the extract as well as for the ascorbic acid. However, beyond a concentration of the order of 0.5 mg / ml, the percentage inhibition remains practically stable. These observations are in agreement with the results reported above and which demonstrate that the anti-radical activity of the sulfated polysaccharides is concentration-dependent [21, 26].

The results show that the CNSP has a notable capacity to trap the DPPH free radical close to that of vitamin C with a maximum inhibition of 82.44%. However, the sweeping effects of DPPH radicals on polysaccharides were relatively lower than that of ascorbic acid at the same concentration. Also, Souza et al. [26] have shown that this synthetic antioxidant has a higher anti-free radical effect than the sulfated polysaccharide of the red alga *Gracilaria birdiae*.

The antioxidant capacity of the CNSP was measured using an electron transfer-based assay. In this study, ABTS was used as a cation-radical oxidant (ABTS^+^), blue-green colored and with absorbance characteristics at 734 nm.

Fig. 2b illustrates an important trapping activity of the ABTS radical for CNSP at dependent concentrations, with ascorbic acid as a reference with inhibition percentages of the order of 87.25% and 91.12%, respectively Allows it to be explored as a natural antioxidant potential.

The purified fraction of the polysaccharide extract isolated from *Dendrobium nobile Lindl* was considered as a potent inhibitor of the ABTS radical with a percentage inhibition of the order of 82.6% at a concentration of 2.0 mg/ml [27], which is in line with our results. In addition, sulfate polysaccharides extracted from *Ulva fasciata, Gloiopeltis furcata, Sargassum henslouianum* have unsatisfactory antioxidant performance to ABTS with inhibitions of 10%, 36.34% and 27.95% respectively [28]. Which were considered low compared to the one demonstrated in our study.

The antioxidant properties of those kinds of polysaccharidic compounds are due to their redox properties, which play an important role in neutralizing and absorbing free radicals, decomposing peroxide or quenching singlet and triple oxygen.

In the other hand, the free radical scavenging activity of CNSP could be related to the inhibition of the generation of free radicals by chelating ions such as ferrous and copper instead of directly scavenging them. Several reports were indicated that the structure of compounds containing more than one of the following functional groups, -OH, -SH, -COOH, -PO3H2, -C=O, -NR2, -S-, and -O-, is in favor of chelating ability [24, 25]. Therefore, presence uronic acid and sulfate groups appeared to be essential in demonstrating the chelating ability of polysaccharides.

According to the findings of Barahona et al., Shao et al. and Li et al. [1, 28, 29], it can be suggested that chemical structure, molecular weight, high sulfate level could have some effect on antioxidant activities of sulfated polysaccharides. But, however, there is a lack of data on the correlation between the structure and functions properties.

### Antibacterial and antifungal activities

Antimicrobial substances are defined as substances used to destroy or inhibit the growth of micro-organisms, including antibiotics and other antibacterial and antifungal agents. However, due to the increasing concern of consumers for foods containing such synthetic additives, the search for natural additives, particularly of marine origin, has in particular increased in recent years.

Our polysaccharide extract was tested in vitro to evaluate its antibacterial and antifungal activity and its ability to stimulate the expression of immune mediators.

The disc-diffusion method is a technique for a preliminary idea of the ability of an extract to inhibit microbial growth, but it cannot give an idea of the concentration of extract necessary to inhibit it. In order to better evaluate this activity a further study was carried out by the determination of the minimum inhibitory concentrations (CMI) of CNSP against the various bacterial strains according to the micro-dilution method.

The antimicrobial activity of the CNSP against the microorganisms analyzed in this study was evaluated qualitatively and quantitatively according to the presence or absence of zones of inhibition, the diameter of the zone (DD) and the minimum inhibitory concentration (MIC), compared to ampicillin and cycloheximide used as reference antibiotic and antifungal drugs (Table 1).

**Table 1:** Antibacterial and antifungal activities of the sulfated polysaccharide from *Cymodocea nodosa* using agar disc diffusion

Strains	DD (CNSP)	DD (Controls)	MIC (mg/ml)
Bacterial strains Gram (−)			
*Escherichia coli*	19 ± 1.1	22 ± 1.0	50
*Escherichia coli* DH5 (alpha)	20 ± 0.5	20.6 ± 0.5	25
*Listeria monocytogene*	18 ± 1.0	21.5 ± 0.7	25
*Salmonella enterica*	21.5 ± 0.7	26 ± 0.6	6.25
*Bacillus subtilis*	18.6 ± 0.5	22 ± 1.4	6.25
*Bacillus amyloliquefaciens*	17 ± 0.5	21 ± 0.9	50
*Agrobacterium tumefaciens*	–	12 ± 1	–
*Pseudomonas aerigunosa*	-	9. 5 ± 0.7	–
Bacterial strains Gram (+)			
*Staphylococus aureus*	23 ± 1.4	25.6 ± 0.5	25
*Micrococcus luteus*	24 ± 0.5	20 ± 0.5	6.25
Fungal strains			
*Aspergilus niger*	15 ± 1.4	21 ± 1.0	6.25
*Saccharomyces cerevisiae*	–	25.6 ± 0.5	–
*Fusarium oxysporum*	14.3 ± 1.5	20 ± 1.5	12.5
*Candida albicans*	18 ± 1.0	26 ± 0.5	12.5

Values are expressed as mean ± standard deviation (*n* = 3)

DD (CNSP): Disc Diameter of inhibition (halo size) in (mm) of CNSP 100 μg/disc,

DD (controls): Disc Diameter of inhibition zone of ampicillin (10 μg/disc) and cycloheximide (10 μg/disc), were used as positive controls for bacteria and fungi, respectively,

MIC: minimum inhibitory concentration (mg/ml),(−) no activity

**Fig. 3:**
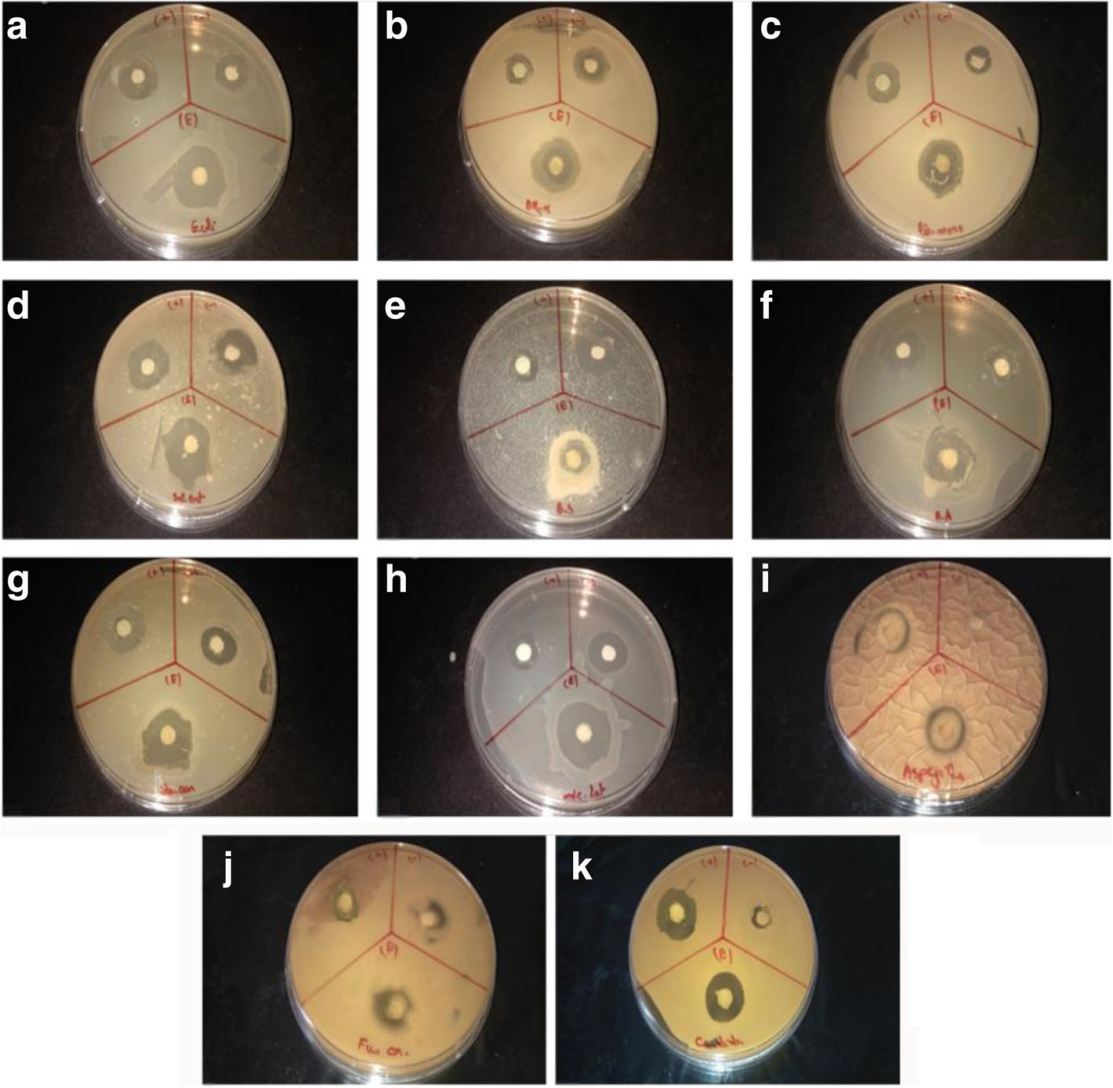
The positive antibacterial and antifungal effects of CNSP against (**a**)*: Escherichia coli, *(**b**)*: Escherichia coli DH5 (alpha), *(**c**)*: Listeria monocytogene, *(**d**)*: Salmonella enteria, *(**e**)*: Bacillus subtilis, *(**f**)*: Bacillus amyloliquefaciens, *(**g**)*: Staphylococus aureus, *(**h**)*: Micrococcus luteus, *(**i**)*: Aspergilus niger, *(**j**)*: Fusarium oxysporum, *(**k**)*: Candida albicans*

It should be noted that the highest level of activity marked by the CNSP was recorded against Gram (+) *Micrococcus luteus* and *Staphylococcus aureus* bacteria with an inhibition diameter of the order of 24 mm and 23 mm, followed by activities Slightly less important or absent for Gram (−) bacteria (Fig. 3).

The results revealed that Gram^+^ bacteria were more sensitive to CNSP than Gram(−) with MIC values ranging from 6.25 to 50 mg/ml. This is in line with recent studies on other marine sulfated polysaccharides isolated from the skin of squid (*Loligo vulgaris*) and fish (*gray triggerfish and smoothhound*) and other marine species (*Sargassum swartzii*) Which showed marked antibacterial activities against both types of bacteria with a high inhibition against Gram (+) [2, 21] having MIC similar to those reported in this study.

The high sensitivity of *Staphylococcus aureus* and *Micrococcus luteus* could be due to the structure of the outer membrane and cell wall. The resistance of CNSP to Gram (−) bacteria could probably be attributed to their outer membranes that surround the cell wall and limit the diffusion of hydrophobic compounds by the lipopolysaccharides.

Like all therapeutic antibiotics polysaccharides CNSP work by binding to the 30S subunit of bacterial ribosomes. This interaction interferes with the translation of messenger RNAs by inducing errors in codon decoding. These errors cause the translation to stop or the synthesis of truncated, aberrant or inactive proteins. The inability to synthesize normal and functional proteins causes the death of bacteria.

On the other hand, for Gram (+) bacteria, the absence of this barrier allows the direct contact of the CNSP components with the bilayer phospholipids of the cell membrane, which causes an increase in ion permeability and passage of constituents or an alteration of vital intracellular bacterial enzymatic systems [30].

Concerning the antifungal activity of the CNSP, the zone of maximum inhibition was observed against *Candida tropicalis* (18 mm) followed by *Aspergilus Niger* (15 mm) and *Fusarium oxysporum* (14.3 mm) with no activity for *Saccharomyces cerevisiae* (Table 1).

In fact, the work described by Ramasamy et al. [31] showed that the polysaccharide extract of the *Sepiaprashadi cuttle* fish exhibited an interesting antifungal activity against *Aspergillus fumigatus*, *Aspergillus flavus* and *Rhizopussp* with no marked activity against *Candida sp*. However, sulfated polysaccharides in the calamar skin (*Loligo vulgaris*) showed significant antifungal activities against *Alternaria solani, Botrytis cinerea and Fusarium solania* with inhibition diameters of 23, 12 and 11 mm respectively [2].

In addition, the positive controls used in this study such as cycloheximide and ampicillin have a higher activity than the polysaccharide tested. This could be explained by the fact that pure components, such as antibiotics and antifungals, give an antimicrobial activity that is more potent than a complex mixture such as the polysaccharide studied.

Therefore, further study will be required on the purification of the active ingredient of this extract in order to open the way for the development of new potential drugs for the treatment of resistant opportunistic fungal infections.

### Antiproliferative activity of CNSP

The natural cytotoxic and anti-tumor substances are substances with aggressive activity against certain cells which are thus lysed or killed. They tend to fight against the tumor, especially they aim at its destruction or reduction. The tumor is defined as any neo-formation tissue with respect to the normal homologous tissue at the expense of which it has developed, which tends to persist and increase and which escapes the biological rules of cell growth and differentiation.

**Fig. 4:**
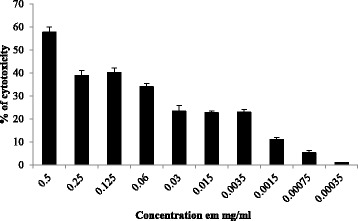
The anti-proliferative activity and percentage of cytotoxicity of CNSP extract on Hela cells

In this section, we aim to measure the cytotoxic activity of CNSP at different concentrations on human cancer cultures (Hela cell from a cervical tumor) performed with the MTT test for 72 h with increasing doses of product (Fig. 4).

This in vitro study showed that our sulfated polysaccharide extracted from the leaves of *Cymodocea nodosa* is a potential cytotoxic compound with these different concentrations.

The results revealed that 0.5 mg/ml of CNSP had the highest cytotoxicity percentage in the order of 58.33 ± 2.13 on Hela cells.

These results are in agreement with those of Costa et al. [22] which reported that all sulfated polysaccharides extracted from 11 species of marine algae showed antiproliferative activity of Hela cells at a dose dependent. These analyzes showed that there was a significant correlation (R2 = 0.934) between the sulfate content of these polysaccharides and the inhibition of cell proliferation. This indicates that the structural characteristics of a polysaccharide such as degree of sulfation, molecular weight, sulfation position, sugar type and glycosidic branching are factors influencing the cytotoxic effect [32].

In recent years, many sulfated polysaccharides such as ulvans and fucans have inhibited the proliferation of human tumor cells (HL60, sarcoma 180), mouse colon cancer cell lines (CT-26, B-16) And the human leukemia cell line (U-937) [33–35].

FeSO_4_ is a chemical agent that has been chosen to induce an oxidative stress state in the Hela line. H_2_O_2_ is an ERO that is not especially toxic to the cell, but in the presence of a low dose of transition metal anion, hydrogen peroxide can interact with the superoxide anion to produce, according to the fenton reaction, the hydroxyl radical which is very active. FeSO4 produces different types of ERO (superoxide anion, hydrogen peroxide and hydroxyl radical) by reacting the ferrous ion (Fe2^+^) with oxygen. The oxidant effect of these agents was verified by the MDA assay, which is one of the final products of lipid peroxidation. Its chemical structure is characterized by the presence of two aldehyde functions giving it a high reactivity with respect to all biological molecules such as proteins.

**Fig. 5:**
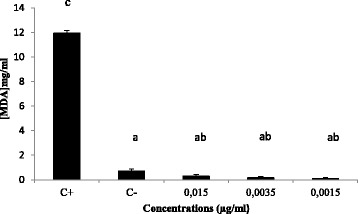
MDA levels in CNSP supplemented Hela cell line. Cells were cultured in 25 cm^2^ flasks with 0.015, 0.0035 and 0.0015 μg/ml of CNSP for 72 h. Oxidative stress was induced by addition of Fe^2+^ to the cells for 1 h at a final concentration of 100 μM. TBARs were compared to untreated cells (C-), cells treated with Fe^2+^ alone (C+), (*p* < 0.05): ^a^ *P* < 0.05 compared with (C+), ^b^ *P* < 0.05 compared with (C-), ^c^ *P* < 0.05 compared with different concentration of CNSP

Following the induction of stress, we observed that Hela cells pretreated with different CNSP concentrations prior to incubation with FeSO_4_ showed a significant reduction in MDA compared to Hela cells treated with FeSO_4_ (*P* < 0.05) (Fig. 5).

Overall, the CNSP inhibits lipid peroxidation in a dose-dependent manner, resulting in a reduction in the MDA level of 11.95 mg/ml for the FeSO_4_ treated cells at 0.10 mg/ml at a concentration of 0.0015 μg /ml. Similar results have been obtained by Zhao et al. [36] who have proved the best inhibitory effect of a polysaccharide isolated from *Ganoderma lucidum* on the high levels of MDA, as well as their size and the complexity of their structure Difficult to study the exact mechanisms of action of polysaccharides.

## Conclusion

The present study is the first to investigate and evaluate the chemical composition, biological properties and nutritional attributes of polysaccharide extracted from the leaves of *Cymodocea nodosa* seagrasses species. The results revealed that CNSP has a number of attractive antioxidant, antimicrobial and cytotoxic properties, which make it a promising candidate for future application in various nutraceutical and functional food industries as well as in alternative medicine and natural therapies. Accordingly, further studies, some of which are currently underway in our laboratories, are needed to explore the correlations between its chemical characteristics and biological activities and investigate its mode and mechanisms of activity as well as to investigate their activities in human subjects.
